# The Tokyo subway sarin attack has long-term effects on survivors: A 10-year study started 5 years after the terrorist incident

**DOI:** 10.1371/journal.pone.0234967

**Published:** 2020-06-23

**Authors:** Aya Sugiyama, Toshihiko Matsuoka, Kazuaki Sakamune, Tomoyuki Akita, Ryosuke Makita, Shinsuke Kimura, Yukio Kuroiwa, Masataka Nagao, Junko Tanaka

**Affiliations:** 1 Department of Epidemiology, Infectious Disease Control and Prevention, Graduate school of Biomedical & Health Sciences, Hiroshima University, Hiroshima, Japan; 2 Department of Forensic Medicine, Graduate school of Biomedical & Health Sciences, Hiroshima University, Hiroshima, Japan; 3 Non-Profit Organization (NPO) Recovery Support Center, Tokyo, Japan; Chiba Daigaku, JAPAN

## Abstract

**Objectives:**

The Tokyo subway sarin attack in 1995 was an unprecedented act of terrorism that killed 13 people and sickened more than 6,000. The long-term somatic and psychological effects on its victims remain unknown.

**Methods:**

We conducted analyses on the self-rating questionnaire collected annually by the Recovery Support Center (RSC) during the period from 2000 to 2009. The RSC is the only organization that has large-scale follow-up data about sarin attack victims. The prevalence of self-reported symptoms was calculated over 10 years. We also evaluated the prevalence of posttraumatic stress response (PTSR), defined as a score ≥ 25 on the Japanese-language version of the Impact of Event Scale–Revised. The multivariate Poisson regression model was applied to estimate the risk ratios of age, gender, and year factor on the prevalence of PTSR.

**Results:**

Subjects were 747 survivors (12% of the total) who responded to the annual questionnaire once or more during the study period. The prevalence of somatic symptoms, especially eye symptoms, was 60–80% and has not decreased. PTSR prevalence was 35.1%, and again there was no change with time. The multivariate Poisson regression model results revealed “old age” and “female” as independent risk factors, but the passage of time did not decrease the risk of PTSR.

**Conclusions:**

Although symptoms in most victims of the Tokyo subway sarin were transient, this large-scale follow-up data analysis revealed that survivors have been suffering from somatic and psychological long-term effects.

## Background

During the morning rush hour on March 20, 1995, the nerve gas sarin (isopropyl methylphosphonofluoridate) was used in a terrorist attack on commuter subway trains in Tokyo, Japan. Tokyo Metropolitan Police Department reported that a total of 13 people were killed, and 6,226 people filed an injury report to the police. This was the largest terrorist attack targeting civilians using a warfare nerve agent in modern times.

Sarin, is an organic phosphorus compound developed for use during conventional warfare in the late 1930s, is a type of nerve gas that acts as a cholinesterase inhibitor. The first symptom after exposure to low air concentrations of nerve gases is miosis. In the case of sarin, this symptom arises in 50% of exposed people at about 3 mg•min/m^3^. Higher exposures cause more significant incapacitation, eventually leading to death for 50% of exposed people at 70–100 mg•min/m^3^ [1].

However, most studies of sarin poisoning have only covered the acute effects, and the long-term effects of sarin have not yet been fully demonstrated [1, 2]. The Recovery Support Center (RSC), a non-profit organization, is the largest organization that has been providing support to the victims of the two Aum Shinrikyo sarin gas attacks; the aforementioned incident in the Tokyo subway in 1995, and a previous one in Matsumoto in 1994 that killed 8 citizens and injured about 660. As one of its supportive measures, the RSC has been conducting annual health checkups on the victims, including a questionnaire survey. Currently, the RSC is the only organization that has large-scale follow-up data on sarin attack victims. To date, a few studies have analyzed the RSC long-term follow-up data [3, 4], Kawana et al. reported chronic posttraumatic stress symptoms in victims from 5 to 8 years after the attack. In this study, to determine the long-term effect of the Tokyo subway sarin attack, we analyzed mental health problems as well as somatic health complaints over a period of 14 years using the RSC’s large-scale cohort data.

### Methods

**Fig 1:**
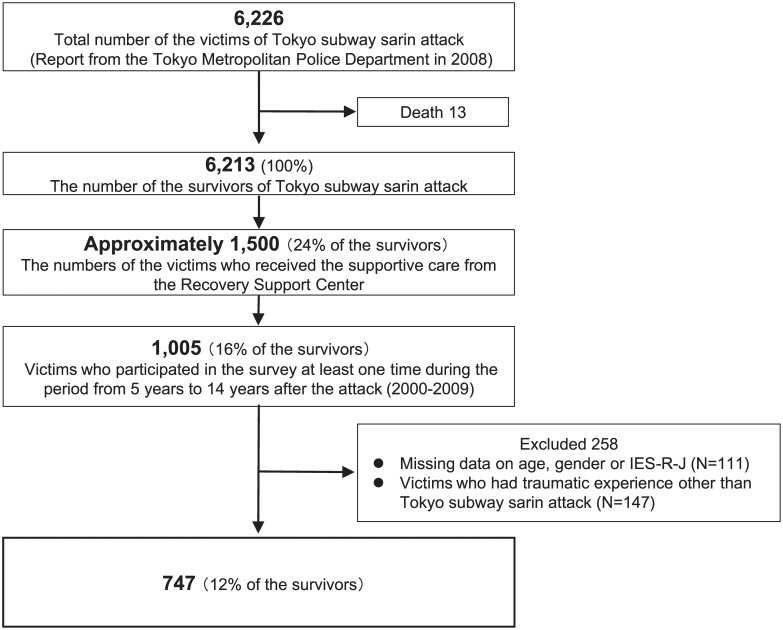
Schematic diagram of subject selection procedure.

Since 2000, 5 years after the Tokyo subway sarin attack, the annual health checkups provided by the RSC have included three kinds of self-rating questionnaires: the basic questionnaire, the 34-item questionnaire for subjective somatic and psychological symptoms, and the Japanese-language version of the Impact of Event Scale–Revised (IES-R-J). The period of this study spans from 2000 to 2009. A schematic diagram of the subject selection procedure is shown in Fig 1. Among the 6,213 survivors, we analyzed 747 victims (12% of the survivors) with no missing data.

In this study, the questionnaire results compiled by the RSC were converted into an anonymized database for analysis. The basic questionnaire included five items including age, gender, subway line, the hospital where the subject received treatment after the attack, and the duration of hospitalization. The 34-item questionnaire for subjective somatic and psychological symptoms was developed by the staff of St. Luke’s International Hospital [5]. The item “numbness in a limb” was added to the survey in 2007, 12 years after the attack. IES-R is a simple and widely distributed self-rating questionnaire for screening for Post-Traumatic Stress Disorder (PTSD) symptoms [6], which was developed by adding some questions to the Impact of Event Scale [7]. The IES-R consists of 22 questions concerning the three main symptoms of PTSD (re-experiencing, avoidance, and hyperarousal). The Japanese-language version of the IES-R (IES-R-J) has been extensively tested for reliability and validity [8]. Based on previous reports [3, 8–11], we defined Posttraumatic stress response (PTSR) as an IES-R-J score of 25 points or higher (out of 88 points possible).

### Data analysis

#### 1. Assessment of 34 subjective somatic and psychological symptoms

The rates of positive responses to the self-rating questionnaire among victims were calculated by year. The 34 subjective symptoms consisted of 14 physical symptoms, eight eye symptoms, and 12 psychological symptoms. Total scores were calculated for each of the physical symptoms, eye symptoms, and psychological symptoms, and their correlation was analyzed by Pearson’s method.

#### 2. Analysis of the prevalence of PTSR (IES-R-J ≥ 25)

The prevalence of PTSR from 5 to 14 years after the attack was calculated. Prevalence by gender was compared by chi-squared test. The 95% confidence intervals (CIs) were estimated based on Wald’s method. Because the annual data included both those who were continuously undergoing checkups and those who were not, we also compared the prevalence at the 14^th^ year for each subgroup, categorized according to the number of previously received health checkups, by chi-squared test.

To evaluate the effects of age, gender, and year factors on the prevalence of PTSR among the victims, we applied the following multivariate Poisson regression model and estimated the risk ratios of each factor by the maximum likelihood method:

$$y_{ijk}\sim \text{Poisson(}\mu_{ijk}),\text{log}(\mu_{ijk})=\text{log}(N_{ijk})+\mu +A_{i}+G_{j}+Y_{k}$$

where *μ*, *A*_*i*_, *G*_*j*_, and *Y*_*k*_ denote intercept, age factor (*i* = 1 for under 29, *i* = 2 for 30s,…, *i* = 5 for 60s and over), gender factor (*j* = 1 for male, and *j* = 2 for female), and year factor (*k* = 1 for 5 years later, *k* = 2 for 6 years later,…, *k* = 10 for 14 years later). *y*_*ijk*_, *μ*_*ijk*_, and *N*_*ijk*_ denote the expected number of patients, real number of patients, and number of population in the *i*-th age, *j*-th gender, and *k*-th year, respectively.

Statistical analyses were performed with software JMP version 9 (SAS Institute, Cary, NC, USA). P values < 0.05 were considered significant.

### Ethical considerations

This was a retrospective observational study using existing data without any intervention or invasion. The existing data were converted into an anonymous database to protect privacy. Based on the low risk to the participants, the study received ethical approval for the use of an opt-out methodology. We announced the detailed information about the content of this study on RSC’s homepage with its contact address, so that the participants who did not want to participate in this study could contact the organization and claim their right to refuse. The Ethical Committees for Epidemiological Research at Hiroshima University waived further informed consent and approved the study with approval number E-323-1.

All methods were performed in accordance with the relevant guidelines and regulations.

## Results

**Fig 2:**
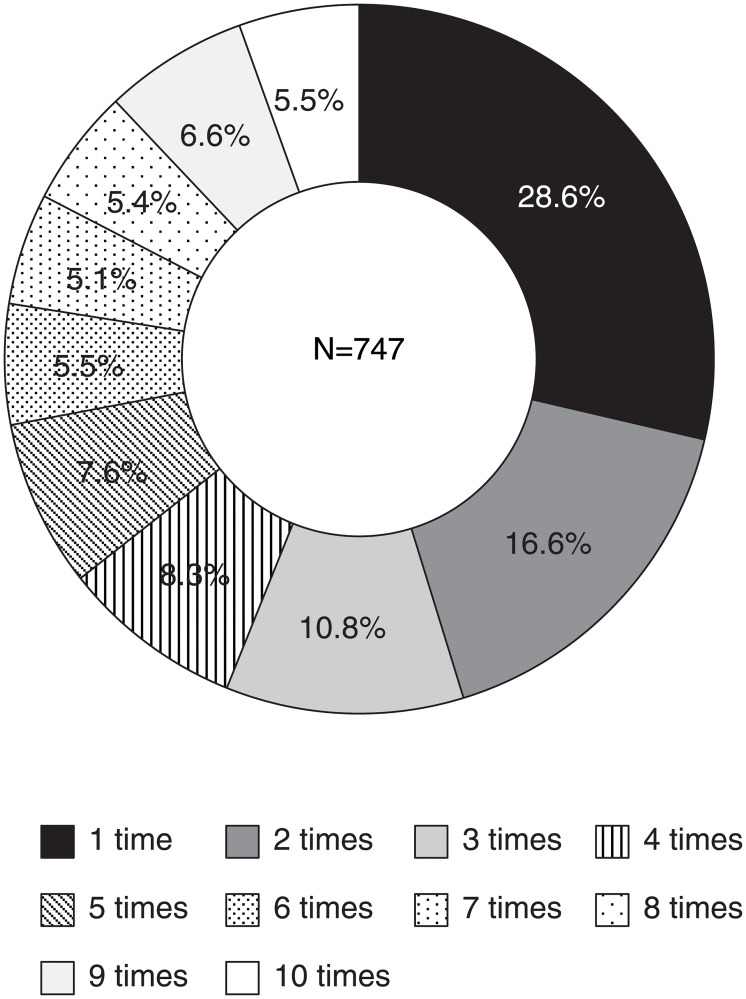
Number of participations in the survey during the period from 5 to 14 years after the attack (2000–2009).

The total number of victims who have participated in the survey once or more during the investigation period was 747 (412 men and 335 women) (Table 1). The average age at the time of the sarin attack was 42.7 ± 12.0 years (14–68 years) for men and 30.3 ± 10.4 years (13–70 years) for women. Age distribution by gender of the subjects analyzed in this study was almost the same as the overall age distribution by gender [11]. Fig 2 shows the distribution of the total number of times that each victim participated in the survey. People who participated only once accounted for the largest number of subjects (28.6%).

**Table 1:** Victims of the Tokyo subway sarin attack who participated in the survey at least one time during the period from 5 to 14 years after the attack (2000–2009).

Years after the attack (year)	N (Male/Female)	Age at exposure (mean±SD)
5 (2000)	431 (231/200)	37.0±13.0
6 (2001)	319 (178/141)	37.9±13.0
7 (2002)	236 (119/117)	36.7±13.3
8 (2003)	313 (166/147)	38.1±13.4
9 (2004)	261 (138/123)	38.7±13.4
10 (2005)	250 (133/117)	38.5±13.4
11 (2006)	264 (142/122)	38.3±13.0
12 (2007)	271 (156/115)	39.0±13.0
13 (2008)	274(150/124)	38.2±13.2
14 (2009)	302(173/129)	38.5±13.4
**Total from 5 to 14 years after the attack (2000–2009)**	**747(412/335)**	**37.0±12.9**

### The 34 subjective somatic and psychological symptoms from 5 to 14 years after the attack

**Fig 3:**
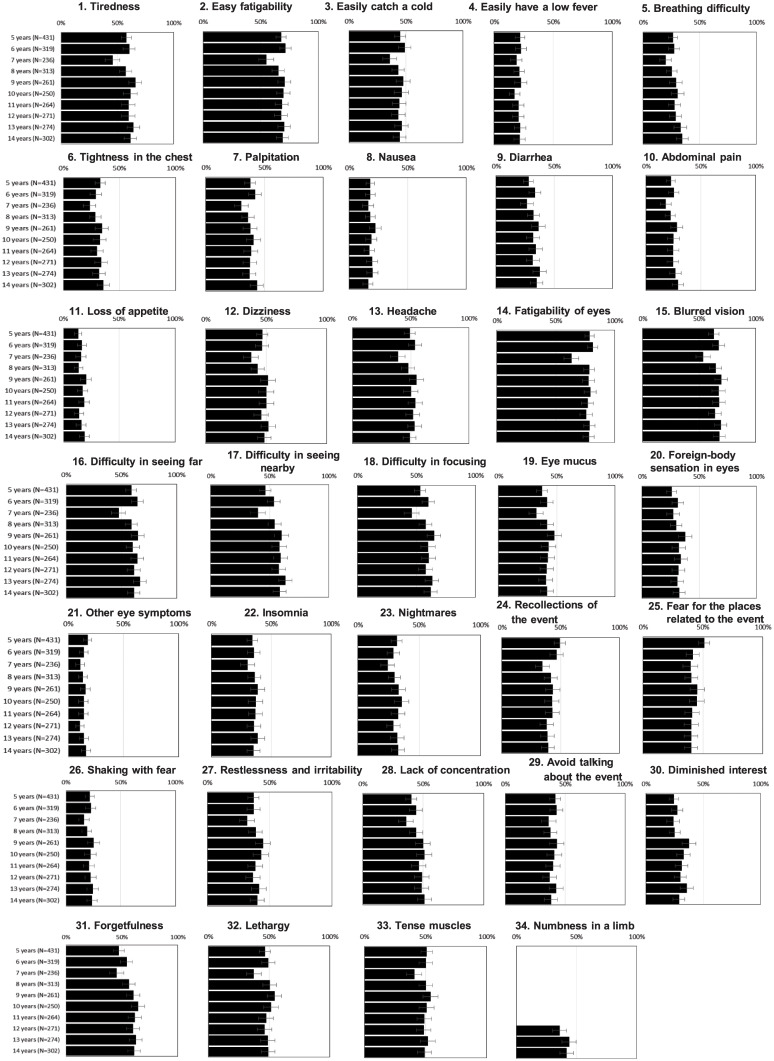
Rates of positive responses on the self-rating questionnaire (34 subjective somatic and psychological symptoms) among victims of the Tokyo subway sarin attack during the period from 5 to 14 years after the attack (2000–2009) (N = 747). Bars indicate 95% CIs.

Based on the results of the self-rating questionnaire about 34 subjective somatic and psychological symptoms, the positive rates for each symptom, by survey year, are shown in Fig 3. For eye symptoms, 79% of the subjects reported “fatigability of eyes” 5 years after the attack, and this value remained as high as 78% 14 years after the attack. The prevalences of “blurred vision” (60–70%) and “difficulty in seeing far,” “difficulty in seeing nearby,” and “difficulty in focusing” (50–60%) remained at almost the same level for 10 years. In regard to somatic symptoms, “easy fatigability” was most common and did not exhibit a downward trend: the incidence was 67.5% at 5 years and 68.9% at 14 years after the attack. The proportion of “headache” and “dizziness” remained around 40–50% over 10 years. The symptom of “numbness in a limb” was added 12 years after the attack, and about 40% of subjects replied affirmatively. In regard to psychological symptoms, the proportion that subjects reporting “recollections of the event” and “fear for the places related to the event” remained at 40% throughout 10 years, and did not exhibit a downward trend over time. Most of the victims stated that the onset timing of symptoms was “after the event.” The psychological symptoms score was significantly correlated with the somatic and eye symptoms scores (correlation coefficients 0.7 and 0.5, respectively; both p < 0.0001).

### Prevalence and relative risk of PTSR (IES-R-J ≥ 25)

**Fig 4:**
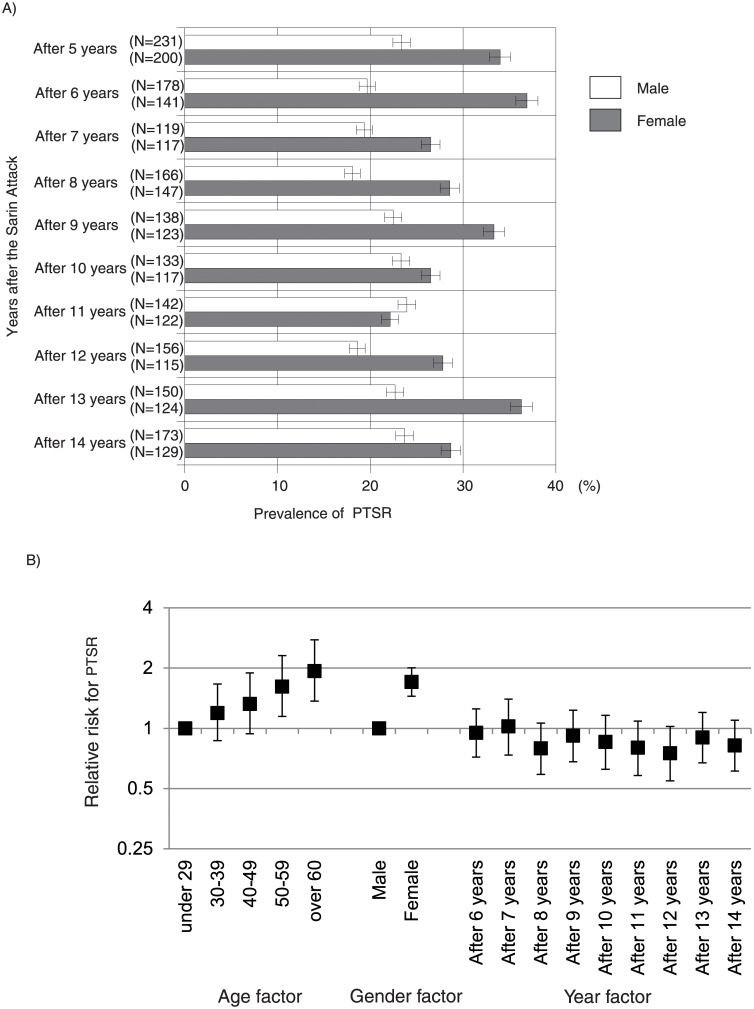
Prevalence and relative risk of PTSR among the victims of the Tokyo subway sarin attack. A) Prevalence of PTSR among the victims of the Tokyo subway sarin attack during the period from 5 to 14 years after the attack (2000–2009). B) Effects of age, gender, and year factors on the risk of PTSR in the victims of the Tokyo subway sarin attack, estimated by multivariate Poisson regression model. Age factor: Age at the time of study. Year factor: Years after the sarin attack. Reference values: age factor, <29 years; gender factor, male; year factor, 5 years after the sarin attack. PTSR: posttraumatic stress response.

The period prevalence of PTSR over 10 years from 5 to 14 years after the attack was 35.1% (95% CI: 33.9–36.2). On a gender basis, the prevalence was 28.4% (95% CI: 27.4–29.4) for men and 43.3% (95% CI: 42.0–44.6) for women (p < 0.0001). The prevalence of PTSR in each survey year is shown in Fig 4A. There was no significant difference in prevalence in the 14^th^ year among subgroups classified according to the number of previously received health checkups (p = 0.2732).

In regard to the risk factors related to the prevalence of PTSR, the effects of age, gender, and year factors are shown in Fig 4B. The relative risk of age, gender, and year factors was adjusted using a multivariate Poisson regression model. The age factor indicated a trend toward higher relative risk at older ages: among people in their 60s, the risk was 1.9 times higher than that in people under 29. The gender factor indicated that females had a 1.7-fold higher risk than males. The year factor revealed no significant change, i.e., the passage of time did not decrease the risk of PTSR.

## Discussion

In this study, we sought to reveal the long-term effects on health of the Tokyo subway sarin attack by analyzing the subjective symptoms of 747 victims, corresponding to about 12% of the total number of victims. Although we were constrained by the fact that data from the first 5 years after the event were not available, we believe that this study helped estimate the long-term health effects on the victims.

The poisoning symptoms that appeared in the most of the victims of the Tokyo subway sarin attack were mild, and the acute poisoning symptoms that appeared in the hospitalized victims included miosis, headache, and visual dimness, in descending order of frequency [12–14]. Full recovery can be expected for those who have experienced mild to moderate exposure to sarin only once [1]. In the case of the Tokyo subway sarin attack, symptoms in most victims reportedly disappeared within 1 month [15–17]. However, the results of this study revealed high percentages of symptoms such as “fatigability of eyes” (80%), “blurred vision” (60–70%), “difficulty in seeing far/nearby” (≥ 50%), and “difficulty in focusing” (≥ 50%) that persisted even 5–14 years after the attack. The prevalence of these symptoms did not decrease over this period. This result is consistent with the results of a large-scale study of victims’ conditions conducted by the National Research Institute of Police Science in 2001, which reported that the symptoms of “fatigability of eyes” and “weakening eyesight” in 910 participants had failed to improve since the time of a previous study in 1998 [18]. Another study reported that miosis, eye movement disorder, reduced accommodation ability, and blepharospasm had persisted in the victims of the Tokyo attack even after a long period of time [19]. These results suggest that sarin toxicity can cause long-lasting eye symptoms.

In regard to somatic symptoms, we observed no decline in “easy fatigability” (60–70%), “tiredness” (~60%), or “headache/dizziness” (~50%), which were reported by a large percentage of subjects. The item “numbness in a limb” was added 12 years after the attack, and about 40% of the subjects reported this symptom. Notably in this regard, distal sensory axonopathy was recognized in some victims after the attack [20], suggesting that sarin toxicity has a long-term effect resulting in peripheral neuropathy.

In regard to psychological symptoms, the rates of “recollections of the event” and “fear for the places related to the event” remained at around 40% over the 10 years. Complaints such as “insomnia” (30–40%), “restlessness and irritability” (30–40%), and “forgetfulness” (50–60%) were also common, and most of these symptoms arose after the attack.

The prevalence of these subjective symptoms was much higher among the victims of the Tokyo subway sarin attack than in the general population. According to a national survey by the Ministry of Health, Labour and Welfare of Japan, the prevalences of these subjective symptoms in Japanese men in their 40s were 2.3% for “blurred vision,” 2.3% for “difficulty in seeing far/nearby,” 4.9% for “tiredness,” 3.1% for “headache,” 1.1% for “dizziness,” 2.3% for “numbness in a limb,” 2.2% for “insomnia,” 2.5% for “irritability,” and 1.3% for “forgetfulness” [21]. Physical symptoms and psychological symptoms were positively correlated in this study. Similar results were obtained in a survey conducted 5–8 years after the attack, and it is possible that somatization has occurred [3].

As for the somatic and psychological subjective symptoms and PTSD in the victims of sarin exposure, some symptoms persisted over the period from 6 months to 5 years after the attack [3, 16, 17, 22–27]. However, there have been few reports of sequelae more than 5 years after the incident [3, 4, 28]. In this study, the prevalence of PTSR was 35.1% and 25.8% at 5 and 14 years after the attack, respectively, significantly higher than the lifetime prevalence of PTSD in average Japanese citizens (1.3%) [29]. Our multivariate Poisson regression analysis with age, gender, and year factors as covariates revealed that the risk of PTSR prevalence did not decrease as time passed after the attack. ‘Old age’ and ‘female’, which also known as risk of depression [30–32], were suggested to be an independent risk of PTSR. Although we cannot provide definitive diagnoses of PTSD because this study was not based on clinical data, the IES-R-J scores that we used are significantly correlated with Clinician-Administered PTSD scale (CAPS), a structured interview method [8], and a cutoff score ≥25 is considered effective for screening for PTSR status [8].

The Aum Shinrikyo incident in Japan is the only case in which civilians have been exposed to sarin. In a military context, soldiers in the Gulf War in the 1990s were exposed to sarin, and veterans complained of multiple health disorders for a long period afterward, consistent with the results of this study. However, pesticide and/or pyridostigmine bromide (PB) are thought to have had a greater effect on Gulf War–related illness than sarin [33].

This study has the following limitations. First of all, the RSC could not support 76% survivors. Since the victims of the Tokyo subway sarin attack was a transient group that only got on the same train and did not have common attributes such as residence or occupation, it was not able to make a list of everyone and invite. Furthermore, data about the first 5 years after the attack were not available in this study. Second, we could not evaluate sarin exposure quantitatively for each victim. Third, all of the evaluated symptoms were subjective symptoms, and we could not evaluate objective findings. Fourth, we could not assess factors such as social class, marital status, and death after the attack, which could have affected the results. Even with such limitations, this study is based on the only long-term follow-up data collected by the RSC, and could therefore help reveal the long-term symptoms that can arise after chemical terrorism, an issue that is not well understood.

## Conclusion

For most victims of the Tokyo subway sarin attack, symptoms were reported to be transient. However, in this analysis of large-scale follow-up data, we found that in many victims, various kinds of somatic and psychological symptoms persisted even after a long period of time has passed. These facts suggested that long-term and periodical care for victims is necessary.

## Supporting information

S1 Data (DOCX)

## Abbreviations

CI: confidence interval
IES-R-J: the Japanese-language version of the Impact of Event Scale–Revised
PTSD: the Post-Traumatic Stress Disorder
PTSR: Posttraumatic stress response
RSC: the Recovery Support Center
SD: Standard deviation
