# Supplementary material for: The Tokyo subway sarin attack has long-term effects on survivors: A 10-year study started 5 years after the terrorist incident
Source: PLoS One. 2020 Jun 23;15(6):e0234967. doi: 10.1371/journal.pone.0234967 (PMC7310687; doi:10.1371/journal.pone.0234967)
Supplement: S1 Data — (DOCX) [file pone.0234967.s001.docx]

Data of victims of the Tokyo subway sarin attack (N=747)*

|  |  | N |
| --- | --- | --- |
| Sex | Male | 412 |
|  | Female | 335 |
| Age | Under 29 | 296 |
|  | 30 | 136 |
|  | 40 | 148 |
|  | 50 | 129 |
|  | Over 60 | 38 |
| Place where encountered the attack | Forgot | 5 |
|  | On the train | 263 |
|  | Out of the train | 156 |
|  | On the train and out of the train | 24 |
|  | Secondary damage | 2 |
|  | No answer | 297 |
| Days of hospitalization at the time of the attack | Forgot | 15 |
|  | More than 8 days | 36 |
|  | 2-7 days | 207 |
|  | Less than 1 day | 72 |
|  | No hospitalization | 168 |
|  | No answer | 249 |

* 747 victims those who participated in the survey at least one time during the period from 5 to 14 years after the attack (2000–2009)

The first time answer for the 34 subjective somatic and mental symptoms among 747 victims of the Tokyo subway sarin attack*

* 747 victims those who participated in the survey at least one time during the period from 5 to 14 years after the attack (2000–2009)

The first time answer for the Japanese version of the Impact of Event Scale-Revised (IESR-J) among 747 victims of the Tokyo subway sarin attack*

* 747 victims those who participated in the survey at least one time during the period from 5 to 14 years after the attack (2000–2009)
